# Effects of Silver Nanoparticles on Multiple Drug-Resistant Strains of *Staphylococcus aureus* and *Pseudomonas aeruginosa* from Mastitis-Infected Goats: An Alternative Approach for Antimicrobial Therapy

**DOI:** 10.3390/ijms18030569

**Published:** 2017-03-06

**Authors:** Yu-Guo Yuan, Qiu-Ling Peng, Sangiliyandi Gurunathan

**Affiliations:** 1 College of Veterinary Medicine/Animal Science and Technology, Yangzhou University, Yangzhou 225009, China; 2 College of Chemistry and Bioengineering, Yichun University, Yichun 336000, China; ygyuan@yzu.edu; 3 Department of Stem cell and Regenerative Biotechnology, Konkuk University, Seoul 143-701, Korea

**Keywords:** silver nanoparticles, *Pseudomonas aeruginosa*, *Staphylococcus aureus*, oxidative stress, metabolic activity, multiple drug resistance

## Abstract

Recently, silver nanoparticles (AgNPs) have been widely used in various applications as antimicrobial agents, anticancer, diagnostics, biomarkers, cell labels, and drug delivery systems for the treatment of various diseases. Microorganisms generally acquire resistance to antibiotics through the course of antibacterial therapy. Multi-drug resistance (MDR) has become a growing problem in the treatment of infectious diseases, and the widespread use of broad-spectrum antibiotics has resulted in the development of antibiotic resistance by numerous human and animal bacterial pathogens. As a result, an increasing number of microorganisms are resistant to multiple antibiotics causing continuing economic losses in dairy farming. Therefore, there is an urgent need for the development of alternative, cost-effective, and efficient antimicrobial agents that overcome antimicrobial resistance. Here, AgNPs synthesized using the bio-molecule quercetin were characterized using various analytical techniques. The synthesized AgNPs were highly spherical in shape and had an average size of 11 nm. We evaluated the efficacy of synthesized AgNPs against two MDR pathogenic bacteria, namely, *Pseudomonas aeruginosa* and *Staphylococcus aureus*, which were isolated from milk samples produced by mastitis-infected goats. The minimum inhibitory concentrations (MICs) of AgNPs against *P. aeruginosa* and *S. aureus* were found to be 1 and 2 μg/mL, respectively. Our findings suggest that AgNPs exert antibacterial effects in a dose- and time-dependent manner. Results from the present study demonstrate that the antibacterial activity of AgNPs is due to the generation of reactive oxygen species (ROS), malondialdehyde (MDA), and leakage of proteins and sugars in bacterial cells. Results of the present study showed that AgNP-treated bacteria had significantly lower lactate dehydrogenase activity (LDH) and lower adenosine triphosphate (ATP) levels compared to the control. Furthermore, AgNP-treated bacteria showed downregulated expression of glutathione (GSH), upregulation of glutathione S-transferase (GST), and downregulation of both superoxide dismutase (SOD) and catalase (CAT). These physiological and biochemical measurements were consistently observed in AgNP-treated bacteria, thereby suggesting that AgNPs can induce bacterial cell death. Thus, the above results represent conclusive findings on the mechanism of action of AgNPs against different types of bacteria. This study also demonstrates the promising use of nanoparticles as antibacterial agents for use in the biotechnology and biomedical industry. Furthermore, this study is the first to propose the mode of action of AgNPs against MDR pathogens isolated from goats infected with subclinical mastitis.

## 1. Introduction

Mastitis is an inflammation of the mammary gland (udder) that causes a chemical and physical reaction in milk produced by goats and cattle. Mastitis is caused by poor hygienic practices in farms and is associated with several viruses, bacteria, and fungi, as well as their toxins. Mastitis is also a multifactorial disease that requires large costs for the treatment of dairy animals. The primary causative agents for mastitis are bacteria, including *Staphylococcus aureus*, *Streptococcus agalactiae*, *Escherichia coli*, *Pseudomonas aeruginosa*, *Corynebacterium bovis*, and *Bacillus cereus*. Mastitis can also be caused a wide spectrum of microorganisms, including fungi, yeast, algae, Chlamydia, and viruses [1,2,3]*.* Mastitis is considered as the most critical threat to the dairy industry because it leads to a significant decline in animal health and milk production, ultimately causing huge economic losses [3]. Gram-negative bacteria cause mastitis due to the presence of the cell wall lipopolysaccharides (LPS), one of the most potent and cell-wall derived immunostimulants, serves as the key virulence factor that elicits the clinical symptoms of mastitis [4,5]. Gram-positive bacteria also produce several important compounds that act as immunostimulators, such as lipoteichoic acid and peptidoglycans, which are responsible for activating immune cells such as monocytes and macrophages to produce inflammatory cytokines and chemokines [6,7,8]. *Pseudomonas aeruginosa* is an important opportunistic pathogen that affects humans, plants, and animals and is often associated with sporadic clinical mastitis. On the other hand, *Staphylococcus aureus* is an opportunist pathogen and a common contaminant and is also a medically important human pathogen capable of causing a variety of infections, ranging from minor skin and wound infections to life-threatening diseases [9].

Antibiotics are the first choice of treatment by veterinarians and farmers and are usually administered to infected animals without identifying the microorganisms responsible for causing mastitis [10]. Antimicrobial therapy is commonly implemented to overcome animal morbidity and mortality due to mastitis. Moreover, broad-spectrum antibiotics comprise the majority of antibiotics that are extensively used against gram-positive and gram-negative bacteria [11]. Unfortunately, the use of antibiotics often fails to eliminate disease antimicrobial resistance (AMR) [12]. Additionally, AMR in bacteria is a public health hazard, and antimicrobial use is considered a potentially important driver of AMR. For example, cloxacillin, an antimicrobial agent similar to methicillin/oxacillin, is extensively used in dry cow therapy [10]. Antimicrobial therapy may be necessary for decreasing morbidity and mortality rates against infectious diseases [13], but also the cause for the emergence and spread of AMR. The increased use of antimicrobials has led to a global increase in the number of antimicrobial-resistant microbes without improving the outcome of treatment [14]. Therefore, the use of antibiotics in dairy farming contributes to increased antimicrobial resistance and represents a major barrier for the treatment of mastitis.

The widespread increase in the use of conventional antimicrobial agents has led to global concerns, such as the rapid emergence of antimicrobial resistant strains, prolonged infection treatment, and increased mortality risk [15,16]. Some conventional antimicrobial agents are unable to cross certain cell membranes, rendering them ineffective for the treatment of intracellular pathogens [15]. Furthermore, the prevalent use of antimicrobial agents in clinical settings and dairy agriculture has led to several problems, including increased risk of microbial resistance, weaker antimicrobial activities, more tedious and prolonged process for monitoring and extending antimicrobial function, and increased difficulty in functioning in a dynamic environment. Therefore, there is an urgent need to overcome the limitations of conventional antibiotics. Recently, advancements in nanotechnology have led to the development of nanoparticles with unique physiochemical properties and functionalization and are able to overcome restrictions posed by conventional antimicrobial agents [15,16]. Hence, simple, environmentally friendly, cost-effective, biocompatible, and long-term antibacterial particles represent a promising solution for use in medicine and farming [17]. Recently, the use of nanomaterials, particularly AgNPs, to complement antibiotics has increasingly gained attention in the academe, industry, and the field of nanomedicine. For instance, nanoparticles with sizes less than or equal to 10 nm exhibit high reactivity with other substances without the need for complementary energy input [18]. Additionally, bacteria are less likely to develop resistance against Ag than against conventional antibiotics [19]. AgNPs have been widely used as antimicrobial agents against bacteria, fungi, and viruses [20,21]. AgNPs were reported as effective antibacterial agents against various gram-negative and gram-positive bacteria [22]. Furthermore, low concentrations of AgNPs and antibiotics were found to be highly effective in inducing loss of cell viability in both gram-negative and gram-positive bacteria [23]. As a result, there is a need to devise methods for the synthesis of AgNPs using various biomolecules (e.g., bacteria, fungi, plant extracts, and small molecules) as templates, particularly via an environmentally friendly process [24,25,26,27]. Quercetin belongs to the class of flavonoids, which are well known polyphenolic compounds widely distributed in fruits, and furthermore have anti-oxidant properties. In addition, quercetin has the properties of electron donation or hydrogen atom donation from the free hydroxyl groups. Quercetin easily and quickly reduces silver ions and can also act as capping agents to prevent the agglomeration of AgNPs. Furthermore, due to unique and potential properties of quercetin, we selected quercetin as a reducing agent for synthesis of AgNPs.

Several studies have proposed that AgNPs could induce cell death in *E. coli*, a gram-negative bacterium, by creating “pits” in the cell wall, eventually leading to increased membrane permeability and inactivation of the respiratory chain [28,29]. On the other hand, the Ag^+^ ion can also inhibit and disrupt protein structure by binding to thiol and amino groups in the cell wall. Recent studies have provided strong evidence that AgNPs induce cell death through ROS generation in various bacteria, including *Pseudomonas aeruginosa*, *Shigella flexneri*, *Staphylococcus aureus*, *Streptococcus pneumonia*, *Helicobacter pylori*, *Helicobacter felis*, *E. coli*, *K. pneumoniae*, and *Bacillus subtilis* [30,31,32]. Although the effects of AgNPs in vitro have been reported, no study has investigated the potential use of AgNPs in eliminating mastitis-causing bacteria in goats. Therefore, the first objective of the present study is the synthesis and characterization of AgNPs using the bio-molecule quercetin as a reducing and stabilizing agent. The second aim of the study is to isolate and characterize predominant isolates from milk samples. The third objective is to evaluate the effect of biologically synthesized AgNPs on representative multiple drug-resistant gram-negative and gram-positive bacteria, namely, *Pseudomonas aeruginosa* and *Staphylococcus aureus*, respectively. Finally, we investigated the mechanism by which AgNPs induce bacterial cell death using various cellular assays.

## 2. Results and Discussion

### 2.1. AgNPs Synthesis Using Quercetin and AgNPs Characterization

**Figure 1:**
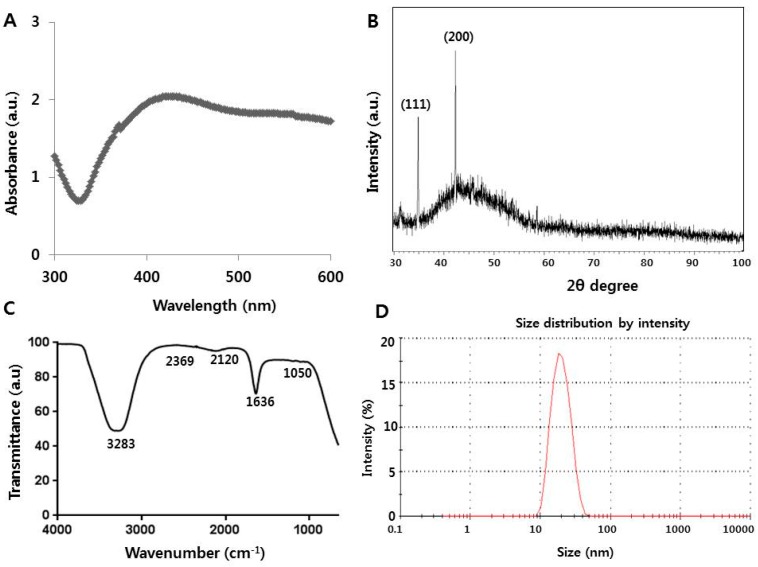

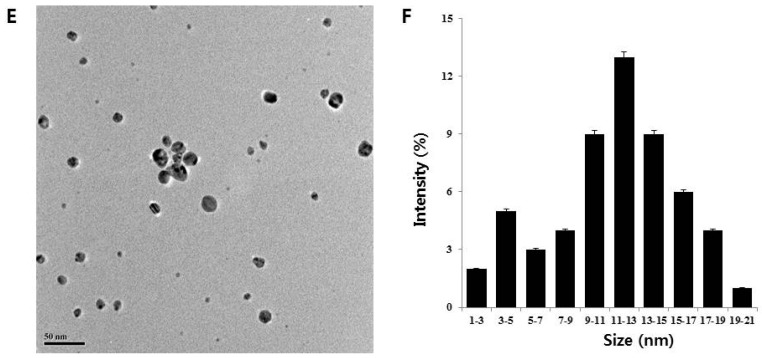
Synthesis and characterization of silver nanoparticles (AgNPs) using quercetin: (**A**) absorption spectrum of AgNPs synthesized using quercetin (**B**) X-ray diffraction spectra of AgNPs; (**C**) Fourier transform infrared spectra of AgNPs; (**D**) size distribution of AgNPs based on dynamic light scattering (DLS); (**E**) TEM images of AgNPs; and (**F**) several fields were used to measure AgNPs particle size; micrograph shows AgNPs with sizes ranging from 1 to 21 nm based on TEM images.

Overuse or misuse of antimicrobials has led to the development of multi-drug resistant bacteria. To overcome the limitations of conventional synthetic antimicrobial compounds, nanotechnology represents an alternative strategy in developing alternative antimicrobial agents that can efficiently kill bacterial cells and display immense potential for use in both medical and veterinary applications. To explore the potential practical use of AgNPs, we prepared the AgNPs nanoparticles using bio-molecule called quercetin. Quercetin belongs to family of flavonols that contain five hydroxyl groups in positions 3, 5, 7, 3′, and 4′ and a carbonyl group in the fourth position. Quercetin acts as an effective and natural free radical scavenger that easily forms complexes with various metals [33]. AgNPs were prepared by mixing 0.5 mg/mL quercetin and 1 mM AgNO_3_ and incubating the resulting mixture at 40 °C for 1 h. The synthesis of AgNPs was determined based on the color change from yellow to dark brown [24]. However, the degree of color change depends on the size of the particles produced in the reaction mixture. Further characterization of AgNPs was carried out using UV–Vis spectroscopy. The absorption spectra showed a maximum peak at 420 nm, which represents the characteristic peak for AgNPs (Figure 1A). Our results are consistent with previous report by Mittal et al. (2014) [34], who utilized quercetin as a reducing and stabilizing agent for AgNPs synthesis. Next, we examined the crystal structure of AgNPs using X-ray diffraction (XRD). The obtained XRD pattern of the synthesized AgNPs was further confirmed by the presence of a characteristic peak in the XRD image (Figure 1B). XRD spectra showed that the crystalline structure of AgNPs has two distinct diffraction peaks at 35.28° and 44.33° corresponding to lattice plane value indexed at (111), and (200), planes of face centered cubic structure silver. These results are consistent with the properties of AgNPs previously prepared using various biomolecules, such as culture supernatants of *Bacillus licheniformis*, *E. coli*, purified fibrinolytic URAK, and quercetin [34,35,36,37]. Further validation was carried out using Fourier transform infrared (FTIR) spectroscopy, which is useful for determining the chemical composition of reactants involved in the synthesis and coating of AgNPs. As shown in Figure 1C, three main bands were observed. The broad band appearing at 3283 cm^−1^ is assigned to O–H stretching vibration and indicates the presence of hydroxyl groups in the reducing agent [38,39] and polyhydroxy groups present in the flavonoids. The peak at 1636 cm^−1^ represents amines. The peak at 2120 cm^−1^ indicates carboxylic acid, and bands at 1050 cm^−1^ indicate the presence of C–O stretching alcohols, carboxylic acids, esters, and ethers. FTIR spectroscopy results confirm that the quercetin can act as both a reducing and stabilizing agent for silver nanoparticles. The toxicity of AgNPs depends on the size of the particles, particularly the particle size in solution. Therefore, we measured the size of AgNPs using dynamic light scattering in solution. The AgNPs used in the present study have sizes ranging from 10 to 50 nm, with an average size of 20 nm (Figure 1D). Finally, we employed transmission electron microscopy (TEM) to determine the size and morphology of AgNPs synthesized using quercetin. TEM images showed that the synthesized AgNPs were well-dispersed and highly spherical in shape with an average size of 11 nm (Figure 1E). We measured at least 200 particles from several TEM images, which also showed the smaller sizes than those measured based on dynamic light scattering (DLS) (Figure 1F). The reason for slightly larger size of the particle in DLS analysis is due to Brownian motion in the solution based analysis. The prepared AgNPs exhibited more desired sizes compared to the synthesized nanoparticles previously reported by Mittal et al. [34], who observed that AgNPs synthesized using flavonoid and phenolics had sizes ranging between of 30 and 35 nm. As we reported earlier, the particle size can be controlled by adjusting factors such as temperature, pH, AgNO_3_ concentration, and concentration of precursor reactants [36]*.* However, smaller AgNPs are considered more effective than larger particles because of higher surface area and more effective binding to the bacterial membrane. Table 1 shows DLS analysis and zeta potential of AgNPs in water and MHB media.

### 2.2. Isolation, Identification, and Characterization of Bacteria from Mastitis-Infected Samples

First, we aimed to determine the prevalence of mastitis in dairy goats reared under field conditions from various provinces in China. Screening of collected milk samples using the Whiteside mastitis test showed that 50% (50/100) of samples were positive for sub-clinical disease. Bacteriological analyses were performed on 50 milk samples, out of which 80% were culture-negative and 20% were culture-positive. Analysis of 150 bacterial isolates collected from the positive milk samples showed that the predominant bacteria was *S. aureus* (52.5%), followed by *P. aeruginosa* (30%), *E. coli* (7.5%), *Bacillus* species (5.5%), and *Corynebacterium* spp. (4.5%). Similarly, Najeep et al. (2013) [40] reported that the most abundant bacterial species in mastitis samples was *Staphylococcus aureus* (61.64), followed by *E. coli* (10.96), *Streptococcus* spp. (9.59), *Pseudomonas/Bacillus* spp*.* (6.85), and *Corynebacterium* spp. (4.11). Ali et al. (2010) [41] reported that *S. aureus* represents one of most frequent etiological agents (45.34%) responsible for dairy goat mastitis cases. Microbial analysis conducted by Scaccabarozzi et al. (2015) [42] demonstrated that *P. aeruginosa* was the most predominant bacterium in mastitis-infected goats. Altogether, our data agree with previous findings and indicate that *S. aureus* and *P. aeruginosa* represent the most dominant bacterial isolates found in mastitis-infected samples from various provinces in China.

### 2.3. Isolation of Multiple Drug-Resistant Bacteria

In vitro antibiotic sensitivity test was performed on all bacterial isolates as described in the Materials and Methods Section. *S. aureus* was found to be most resistant to methicillin (80%), followed by vancomycin (70%), amoxicillin (60.0%), streptomycin (40.4%), tetracycline (40.5%), lincomycin (35.0%), erythromycin (31%), rifampicin (15%), oxacillin (16.7%), norfloxacin (15.8%), doxycicline (13.0%), ciprofloxacin (8.5%), enrofloxacin (8.1%) and gentamycin (7.62%). *Pseudomonas aeruginosa* was found to be most resistant to β-lactams (90%), followed by erythromycin (85%), tetracycline (80%), meropenem (70%), bacitracin (60%), amikacin (35%), gentamicin (30%), ciprofloxacin (20%), ceftazidime (30%), tobramycin (10%), and imipinem (10%). The results demonstrate that both *S. aureus* and *P. aeruginosa* are resistant to more than two different antibiotic classes and are thus declared as MDR. Thus, only these two isolates were considered in further experiments to evaluate the impact of AgNPs on MDR bacteria in mastitis-infected goats.

### 2.4. MIC Determination of AgNPs

To determine the lowest concentration that can completely inhibit the visible growth of bacteria, *P. aeruginosa* and *S. aureus* were treated with increasing concentrations of AgNPs and incubated for 24 h at 37 °C. Our results confirmed that AgNPs inhibit bacteria in a dose-dependent manner. Previously reported MIC values of AgNPs against *P. aeruginosa* and *S. aureus* were found to be 0.59 and 0.75 μg/mL using AgNPs with an average size of 5 nm and synthesized using *Allophylus cobbe* plant extract [23]. Another study reported that bamboo charcoal/silver composites had a MIC value of 3 μg/mL against *P. aeruginosa* [43]. Kora and Arunachalam (2011) [44] reported effective growth inhibition of *P. aeruginosa* at a very low concentration of 2 μg/mL using AgNPs with an average size of 30 nm. In the present study, as shown in Table 2, the synthesized AgNPs have MICs of 1 and 2 μg/mL and MBCs of 2 and 4 μg/mL against *P. aeruginosa* and *S. aureus*, respectively (Table 2). The synthesized nanoparticles have an average size of 11 nm, which is comparable to nanoparticles synthesized in previous studies. In addition, the toxicity of AgNPs was dependent on the particle size and the initial bacterial counts of the inocula. As expected, AgNPs were more effective against the gram-negative *P. aeruginosa* than *S. aureus*, which could be explained by differences in membrane structure and the cell wall composition, which influence bacterial accessibility to AgNPs [22]. The cell walls of both gram-positive and gram-negative bacteria have an overall negative charge because of the presence of teichoic acids and lipopolysaccharides, respectively [22].

### 2.5. Dose- and Time-Dependent Effects of AgNPs against P. aeruginosa and S. aureus

**Figure 2:**
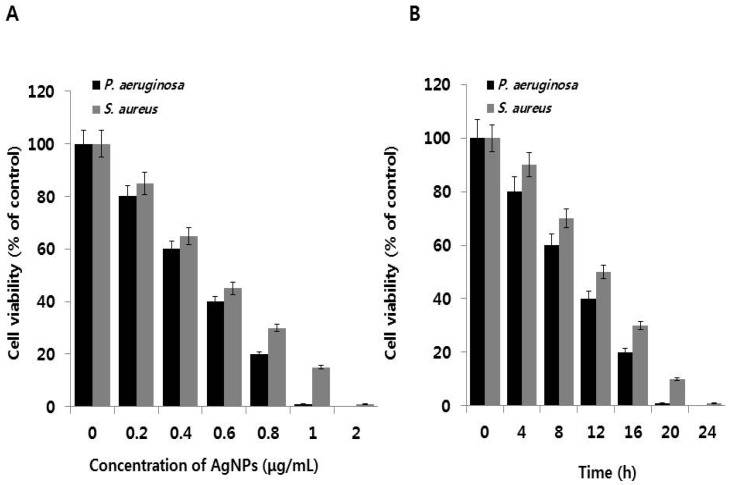
Effect of AgNPs on cell viability: (**A**) *P. aeruginosa* and *S. aureus* cells were incubated with various concentrations of AgNPs. Bacterial survival was determined at 24 h based on a CFU count assay; (**B**) Time dependent effect of AgNPs on *P. aeruginosa* and *S. aureus*. The experiment was performed with various controls, including a positive control (AgNPs and MHB, without inoculum) and a negative control (MHB and inoculum, without AgNPs). Results are expressed as the means ± SD of three separate experiments, with three replicates per experiment. Statistically significant differences between treatment and control groups were determined using student’s *t*-test (*p* < 0.05).

The dose-dependent bactericidal activity of AgNPs was determined in selected MDR strains, namely, *P. aeruginosa* and *S. aureus*. Figure 2A shows the inhibitory effect of biologically synthesized AgNPs (11 nm) at concentrations ranging from 0.2 to 2 µg/mL against *P. aeruginosa* and *S. aureus*. Treatment with AgNPs clearly decreased cell viability in both bacterial strains compared to the control. In *P. aeruginosa*, complete growth inhibition was observed at 1 μg/mL AgNPs, whereas *S. aureus* showed complete inhibition at 2 μg/mL. Cell viability was reduced at higher concentrations of AgNPs, clearly demonstrating that AgNPs inhibits bacterial cell growth in a dose-dependent manner. Furthermore, these results are consistent with the obtained MIC values for each bacterial strain. Interestingly, AgNPs produced via quercetin-mediated synthesis displayed toxicity for both strains. At 0.2 μg/mL AgNPs, delayed growth was observed; at higher concentrations, cell viability was completely inhibited compared to controls. Kalishwaralal et al. [38] reported that 100 nM AgNPs completely inhibited biofilm formation in *P. aeruginosa* and *S. epidermidis*. Li et al. [45] and Anthony et al. [31] reported that 10 μg/mL AgNPs could completely inhibit *E. coli* growth in liquid Mueller Hinton Broth. In addition, the MIC of AgNPs against methicillin-resistant *S. aureus* spp. was found to be 5.6 μg/mL [46]. Das et al. [47] reported that AgNPs prepared using *Ocimum gratissimum* leaf extracts had an average size of 16–18 nm and a MIC of 8 μg/mL. In the present study, AgNPs synthesized using quercetin had smaller sizes and thus can exert more potent bactericidal effects than larger particles because of easier cellular uptake and larger surface are. Smaller particle size also allows stronger interactions with sulfur and nitrogen groups found in the bacterial cell wall and membranes.

The minimum time necessary to reach an inhibitory or bactericidal effect of AgNPs was examined in *P. aeruginosa* and *S. aureus*. We selected respective MIC values for each bacterial strain. Cell viabilities of *P. aeruginosa* and *S. aureus* strains upon treatment with AgNPs are presented in Figure 2B. The time-dependent growth inhibition was determined in the presence of AgNPs for 24 h. In control cultures without AgNPs, bacteria reached the stationary growth phase after 18 h of incubation. Addition of AgNPs resulted in time-dependent inhibition of bacterial growth (Figure 2B). Bactericidal activity of AgNPs gradually increased from 4 to 24 h at their respective MIC concentrations for both strains and eventually reached complete growth inhibition. AgNPs showed a time-dependent and rapid bactericidal activity against *P. aeruginosa* than *S. aureus*. Complete inhibition of *P. aeruginosa* was observed within 20 h, whereas *S. aureus* showed 90% inhibition of cell viability and complete growth inhibition at 24 h. The above results also indicate that the effectiveness of AgNPs treatment is highly dependent on the bacterial cell wall composition. Collectively, our studies suggest that quercetin-assisted synthesis of AgNPs produces particles that effectively inhibit bacterial growth in a dose- and time-dependent manner. These results are consistent with a previous study by Yoon et al. [48], who showed that nanoparticles with sizes ranging from 10 to 12 nm exhibited stronger antimicrobial effect because of larger surface area.

### 2.6. Effect of AgNPs on Metabolic Activity

**Figure 3:**
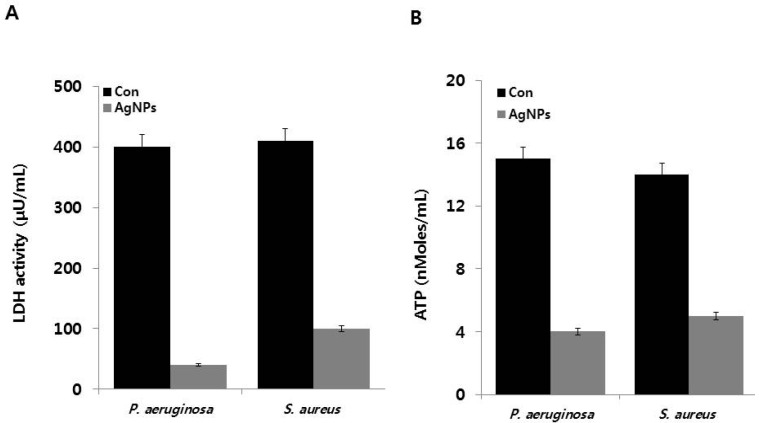
Effects of AgNPs on metabolic activity: (**A**) *P. aeruginosa* and *S. aureus* cells were incubated with respective MIC of AgNPs for 12 h. LDH activity was determined by measuring the reduction of NAD^+^ to NADH and H^+^ during the oxidation of lactate to pyruvate; (**B**) ATP levels were determined by measuring luminescence levels and comparing against an ATP standard curve. Results are expressed as the means ± SD of three separate experiments, with three replicates per experiment. Statistically significant differences between treatment and control groups were determined using student’s *t*-test (*p* < 0.05).

To determine the effect of AgNPs on oxidative stress-induced damage in cell respiration, we measured LDH activity, a reliable marker for determining cell status under oxidative and heat stress conditions [49]. The effects of AgNPs on LDH activities of *P. aeruginosa* and *S. aureus* are shown in Figure 3A. *P. aeruginosa* cells in the control group and AgNP-treated cells showed LDH activities of 400 and 40 μU/mL, respectively. Similarly, the LDH activities of *S. aureus* control cells and AgNP-treated *S. aureus* cells were 410 and 100 μU/mL, respectively. Although both control cells expressed similar LDH levels, the gram-negative bacterium *P. aeruginosa* was found to be more susceptible to AgNPs treatment compared to the gram-positive bacterium *S. aureus*. These results suggest that gram-negative bacteria are more susceptible to cell death than gram-positive bacteria because of their cell wall structure. Our results clearly demonstrated that the activities of respiratory chain dehydrogenases in both *P. aeruginosa* and *S. aureus* were inhibited by AgNPs, consistent with the mechanism of action proposed by Holt and Bard, Kim et al., Li et al., and Soo-Hwan et al. [45,50,51,52]. AgNPs can enter the cells and pass through the outer membrane, peptidoglycan, and periplasm, where they destroy respiratory chain dehydrogenases and alter dissolved oxygen levels in culture [50]. Furthermore, Ag^+^ can interact with the thiol (–SH) group of cysteine by displacing the hydrogen atom to form –S–Ag, thereby suppressing the enzymatic function of affected protein and inhibiting *E. coli* growth [45,52,53,54]. Results of the present study also provide evidence that AgNPs can enter the cells and damage the bacterial cell membrane, which in turn inhibits the activity of some membranous enzymes and results in cell death in both gram-negative and gram-positive bacteria. The inactivation and inhibition of LDH leads to increased leakage of proteins and other macromolecules.

Subsequently, we measured ATP levels in AgNP-treated *P. aeruginosa* and *S. aureus*. ATP is a vital molecule required for many biological functions, including survival, growth, and replication, and acts as a major signaling molecule [55]. All tested samples from AgNP-treated *P. aeruginosa* and *S. aureus* showed significantly lower ATP levels compared to the control samples (Figure 3B). Overall, AgNP-induced cellular stress significantly affects the metabolic activity of *P. aeruginosa* and *S. aureus* via modulation of ATP synthesis, which ultimately affects bacterial growth and reproduction. Recently, Vardanyan et al. [18] reported that Ag nanoparticles directly affect membranes, specifically FOF1-ATPase activity and H^+^-coupled transport. In addition, Ag nanoparticles can impair H^+^ and K^+^ transport even in the presence of *N*,*N*′-dicyclohexylcarbodiimide, an inhibitor of FOF1. Collectively, findings from the present and other studies suggest that FOF1-ATPase in bacterial membranes is a potential target for Ag nanoparticles. AgNPs can inhibit FOF1-ATPase, which plays a crucial role in cell metabolic processes, including bacterial growth and survival.

### 2.7. AgNP-Induced Leakage of Proteins and Sugars

**Figure 4:**
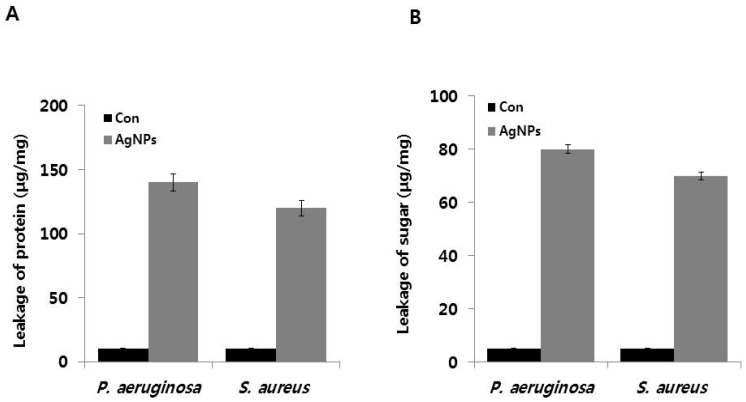
Effect of AgNPs on leakage of protein and sugars: (**A**) for protein leakage analysis, *P. aeruginosa* and *S. aureus* cells were treated with the respective MIC of AgNPs for 12 h, and protein levels were measured using the Bradford assay; and (**B**) for sugar analysis, all test strains were treated with respective MIC of AgNPs for 12 h, and sugar concentrations were measured. Results are expressed as the means ± SD of three separate experiments, with three replicates per experiment. Statistically significant differences between treatment and control groups were determined using student’s *t*-test (*p* < 0.05).

To validate the effects of AgNPs on metabolic activity, we analyzed the levels of intracellular macromolecules such as proteins and sugars. Previous studies have demonstrated that AgNPs cause protein leakage by increasing membrane permeability in bacteria [23] (Gurunathan, 2014). Therefore, we determined the effect of AgNPs on protein leakage by treating *P. aeruginosa* and *S. aureus* cells with AgNPs at their MIC (1 and 2 μg/ mL, respectively) for 12 h. The amounts of protein released in the suspension of the treated cells were estimated using the Bradford assay. Protein leakage was found to be higher in AgNP-treated cells compared to untreated cells (Figure 4A). However, AgNP-treated *P. aeruginosa* showed significantly higher protein leakage (140 μg/mg) compared to *S. aureus* cells (120 μg/mg), suggesting that the gram-positive *S. aureus* had lower antibacterial sensitivity than that of the gram-negative *P. aeruginosa*. Similarly, Soo-Hwan et al. [52] found that protein leakage was significantly higher in *E. coli* than *S. aureus*. Gurunathan et al. [23] showed that protein leakage was significantly higher in the gram-negative bacteria *Escherichia fergusoni* compared to the gram-positive *Streptococcus mutans*. The observed differences in protein leakage could be due to the differences in the structural features of the cell wall, particularly the thickness of the peptidoglycan layer, which functions as a protective barrier against antibacterial agents, such as antibiotics, toxins, chemicals, and degradation enzymes [52]. Altogether, our results were consistent with those of previous studies showing that AgNPs disrupt the bacterial membranes, consequently leading to intracellular leakage of macromolecules [56,57].

To determine the effect of AgNPs on sugar leakage, *P. aeruginosa* and *S. aureus* were treated with 1 and 2 μg/ mL of AgNPs, respectively, for 12 h. As shown in Figure 4B, AgNP-treated cells showed higher membrane leakage of reducing sugars than the untreated control group. Leakage of reducing sugars of AgNP-treated *P. aeruginosa* and *S. aureus* were 80 and 70 μg per 1 mg bacterial dry weight, respectively. However, only controls exhibited only 5 μg/mg leakage, suggesting that AgNPs could induce leakage of reducing sugars from the bacterial cytoplasm. Li et al. [45] reported that *E. coli* cells treated with 10 μg/mL AgNPs for 2 h showed leakage of reducing sugars of up to 102.5 μg per 1 mg of bacterial dry weight, which could be due to the higher concentration of AgNPs. This observation suggests that gram-negative bacteria had significantly higher leakage of proteins and sugars than gram-positive bacteria. Similarly, Gurunathan et al. (2014) [23] demonstrated that *Escherichia fergusoni* had more pronounced AgNP-induced the leakage of sugars than *Streptococcus mutans*. Altogether, these findings clearly indicated that AgNPs exert antibacterial effects by influencing membrane permeability and inducing the leakage of reducing sugars, leading to the bacterial cell death [58]. AgNPs anchor into the cell membrane and enter the cells, leading to osmotic collapse and subsequent release of intracellular materials [45,59]. Collectively, our results suggest that AgNPs decrease membrane permeability of cells and induce the release of intracellular materials, such as sugars and proteins.

### 2.8. AgNP-Induced Oxidative Stress

**Figure 5:**
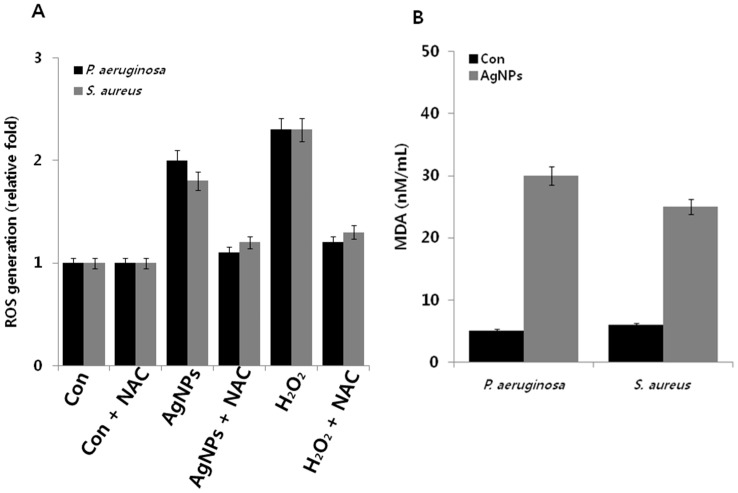
Effect of AgNPs on ROS generation and MDA: (**A**) *P. aeruginosa* and *S. aureus* cells were treated with respective MIC of AgNPs for 12 h. ROS generation was measured using DCFDA; and (**B**) MDA levels were measured using TBARS assay. Results are expressed as the means ± SD of three separate experiments, with three replicates per experiment. Statistically significant differences between treatment and control groups were determined using student’s *t*-test (*p* < 0.05).

Oxidative stress is a condition that results from the imbalance between the cellular levels of pro-oxidants and anti-oxidants and failure in maintaining normal physiological redox-regulated functions [60]. Generally, microorganisms are susceptible to elevated levels of intracellular ROS generated from various natural stresses, such as heat, cold, and toxicants [61]. For instance, various types of antibiotics, including aminoglycosides, β-lactams, and fluoroquinolones, induce ROS generation in bacteria and consequently lead to loss of cell viability [62,63]. Similarly, nanoparticles induce the loss of cell viability in bacteria through ROS generation [16,21,23]. Previous studies strongly suggest that oxidative stress plays a crucial role in mediating toxicity in bacterial cells [64,65]. The interaction between metal nanoparticles and bacterial cells often results in the production of ROS, such as hydroxyl radicals (OH^•^), superoxide ions (O_2_), hydrogen peroxide (H_2_O_2_), and hydroperoxyl radicals, which in turn induce oxidative stress and damage to proteins and nucleic acids [36,66,67]. Therefore, we measured oxidative stress levels in *P. aeruginosa* and *S. aureus* after treatment with AgNPs using the 2′,7′-dichlorofluorescin diacetate (DCFDA) assay. Results suggest that both *P. aeruginosa* and *S. aureus* produced twofold higher levels of ROS compared to untreated cells, whereas *P. aeruginosa* and *S. aureus* treated with H_2_O_2_ (positive control), produced 2.3-fold higher levels of ROS. Interestingly, treatment with NAC (*N*-acetylcysteine), a well-known antioxidant, was found to suppress AgNP-induced ROS generation (Figure 5A). Altogether, the data suggest that ROS generation is a possible mechanism responsible for the antibacterial effects of AgNPs [36,64,65,66,67].

To corroborate the relationship between ROS and MDA content in AgNP-treated *P. aeruginosa* and *S. aureus*, we measured MDA content in treated and untreated cells. Lipid peroxidation of polyunsaturated lipids is one of the most preferred biomarkers for oxidative stress. Lipid peroxidation can produce peroxy radicals and singlet oxygen [68]. Malondialdehyde is a product of lipid peroxidation that can be easily measured in bacterial culture and thus serves as an important biomarker for lipid peroxidation oxidative stress [36,69,70]. In addition, unsaturated aldehydes produced from these reactions have been implicated in modification of cellular proteins and other constituents [71]. Therefore, we measure MDA levels in AgNP-treated *P. aeruginosa* and *S. aureus*. As shown in Figure 5B, MDA levels were significantly higher in both bacterial cultures than in the controls. In addition, MDA levels were sixfold and fivefold higher in AgNP-treated *P. aeruginosa* and *S. aureus*, respectively. These results suggest that the inhibition of bacterial growth was due to AgNP-mediated ROS production. Similarly, *Pseudomonas putida* treated with environmentally relevant concentrations of AgNPs showed increased lipid peroxidation along with suppressed antioxidant defense system [72]. Taken together, these results indicate that AgNPs induce the loss of cell viability through ROS generation, which in turn causes an imbalance between oxidants and antioxidants in cells.

### 2.9. Effect of AgNPs on Antioxidant Levels

**Figure 6:**
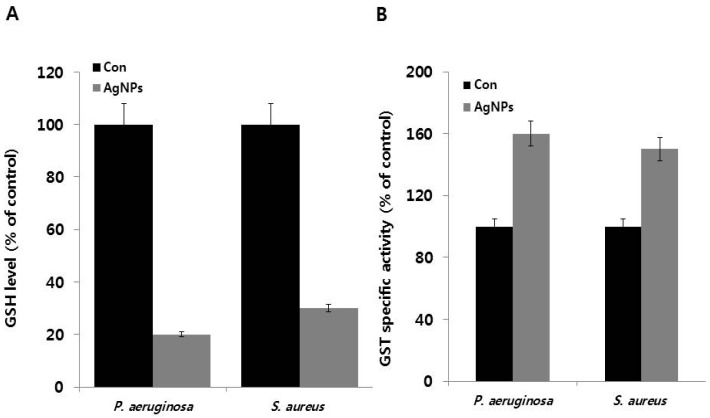
Effect of AgNPs on GSH and GST activity: (**A**) *P. aeruginosa* and *S. aureus* cells were treated with respective MIC of AgNPs for 12 h. GSH levels were measured enzymatically in the clear supernatant based on the reduction of 5,5′-dithiobis-(2-nitrobenzoic acid) by the GSH reductase system; (**B**) GST activity was determined as described in the Materials and Methods Section. Results are expressed as the means ± SD of three separate experiments, with three replicates per experiment. Statistically significant differences between treatment and control groups were determined using student’s *t*-test (*p* < 0.05).

The impact of ROS generation was further analyzed by measuring glutathione (GSH) concentrations and GST-specific activity. In many organisms, glutathione and thioredoxin play important roles in maintaining an intracellular reducing environment and combating oxidative stress in variety of organisms, including gram-positive and gram-negative bacteria. GSH, a thiol-containing tripeptide, predominantly exists at high levels in reduced form and functions by scavenging ROS to maintain balance in the cellular redox environment and protect cells against oxidative stress [73,74,75]. Therefore, we hypothesized that AgNPs induce oxidative stress and influence the levels of cellular antioxidant metabolites such as GSH. The tripeptide GSH and other thiols are major cellular antioxidants [73]. Therefore, we measured GSH levels in AgNP-treated *P. aeruginosa* and *S. aureus*. We observed 80% and 70% decrease in GSH levels in AgNP-treated *P. aeruginosa* and *S. aureus*, respectively, when compared to untreated cells (100%) (Figure 6A). These findings provide evidence that GSH depletion in AgNP-treated cells was responsible for excessive ROS generation, which overwhelmed the antioxidant defense system, leading oxidative stress and loss of cell viability. A similar effect was also observed in *E. coli* cells treated with iodinated chitosan–silver nanoparticle composite [76], as well as *E. coli* and *P. aeruginosa* treated with biologically synthesized AgNPs [67]. Furthermore, Zhang et al. [21] reported similar results in *E. coli* cells exposed to gold nanoparticles and heat stress. Altogether, these above findings suggest that AgNPs exerts negative effects on antioxidant molecules.

To validate the results obtained from GSH assay, further we measured the activity of GST, an enzyme associated with GSH and primarily involved in detoxification processes [77]. Total specific GST activity was measured and represented as percentage over the control. Results revealed a significant difference between the AgNP-treated *P. aeruginosa* and *S. aureus* and untreated cells. *P. aeruginosa* and *S. aureus* showed higher GST activity by 60% and 50%, respectively (Figure 6B). Glutathione reductase (GR) and GST act in concert with the antioxidant compound GSH and play major roles in mediating the tolerance of a *P. aeruginosa* strain to various herbicides [78]. Similarly, *Folsomia candida* exposed to AgNPs displayed decreased levels of GSH and increased GST activity [79]. Exposure of *P. putida* to environmentally relevant concentrations of AgNPs caused ROS production, glutathione depletion, and inactivation of the antioxidant enzymes superoxide dismutase, catalase, and glutathione reductase [72]. Altogether, the above findings suggest that bacteria exposed to AgNPs exhibit increased ROS formation, decreased GSH levels, increased GST enzyme activity, and decreased cell viability, pointing to the role of oxidative stress as a major mechanism of AgNP-induced toxicity in both gram-negative and gram-positive bacteria.

### 2.10. Effect of AgNPs on Superoxide Dismutase (SOD) and Catalase Activity

**Figure 7:**
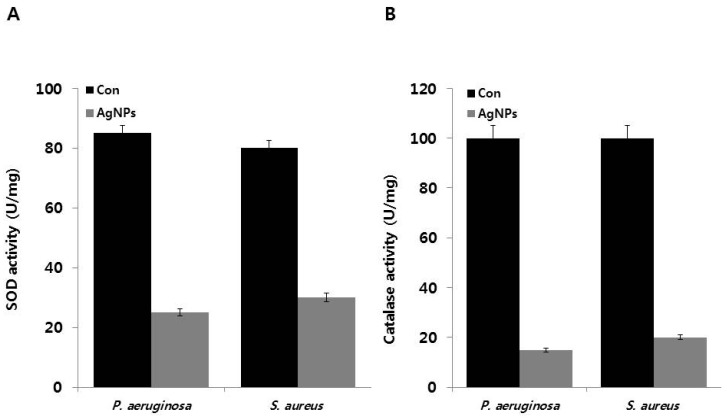
Effect of AgNPs on SOD and CAT activity: (**A**) *P. aeruginosa* and *S. aureus* cells were treated with respective MIC of AgNPs for 12 h. SOD (**A**) and CAT activities (**B**) were measured as described in the Materials and Methods Section. Results are expressed as the means ± SD of three separate experiments, with three replicates per experiment. Statistically significant differences between treatment and control groups were determined using student’s *t*-test (*p* < 0.05).

Silver ions catalyze the decomposition of H_2_O_2_, resulting in the formation of free radicals (OH and/or O_2_) [80,81]. Therefore, the role of ROS generation and its effects on the antioxidant system were evaluated by measuring the activity of ROS-related enzymes, namely, SOD and catalase. To estimate the contribution of antioxidant systems to bacterial response to AgNP-induced oxidative stress, we examined the activity of SOD and CAT in the presence and absence of AgNPs in *P. aeruginosa* and *S. aureus*. The obtained data suggest that AgNP-treated bacteria exhibit significantly lower SOD levels. SOD levels decreased to 25 and 30 U/mg compared to the control (Figure 7A). AgNP-treated cells also showed decreased CAT activity but at slightly higher level than SOD (Figure 7B). Thus, the observed decrease in antioxidant levels in AgNP-treated *P. aeruginosa* and *S. aureus* can be potentially caused by the disruption of the electron transport assemblies in the plasma membrane. Stambe et al. [82] reported that nanoparticle-mediated ROS generation can modulate the antioxidant activities of ROS-metabolizing enzymes, such as NADPH-dependent flavoenzyme, catalase, glutathione peroxidase, and superoxide dismutase. Khan et al. [72] reported that AgNP-treated *P. putida* cells exhibited enhanced lipid peroxidation, accompanied by suppression of the antioxidant defense system. Furthermore, exposure to environmentally relevant concentrations of AgNPs also induced ROS production, glutathione depletion, and inactivation of the enzymes SOD, CAT, and glutathione reductase. Data from our studies and previous studies demonstrate that the differential responses in SOD and CAT activity were due to regulation of enzymatic activity by AgNPs, which increased the susceptibilities of *P. aeruginosa* and *S. aureus* to oxygen radicals. Taken together, AgNPs can act as oxidative stress inducer agents can cause cell death in *P. aeruginosa* and *S. aureus*. Although previous studies have proposed the mechanisms behind the antibacterial activity of AgNPs, the detailed mechanisms of toxicity remain to be elucidated. Many studies have already proven that AgNPs induce cell death in bacteria or multicellular organisms like fungi, plants, and various human cell lines via disruption of the cell envelope, inactivation of respiratory chain enzymes, oxidation of cell components, ROS production, and decomposition of the cellular components [16,22,23,27,32,45,59,64,65,66,67,70,83,84,85]. Based on our results and previous studies, we propose possible mechanisms underlying AgNP-induced cytotoxicity in both *P. aeruginosa* and *S. aureus*. First, AgNPs induce oxidative stress, leading to cell membrane instability and increased membrane permeability by incorporation of AgNPs and forming permeable pits on the membrane, which leads to an osmotic collapse in the cells and releases the intracellular materials. Another possible mechanism is the initial binding of AgNPs with proteins or carbohydrate moieties on the cell surface, leading to cell membrane disruption. In turn, cell membrane disruption causes downregulation of antioxidant molecules like GSH and GST, as well as denaturation of enzymes like SOD and CAT, which impairs the electron transport pathway and leads to cell death [83,86]. As previously suggested, AgNPs can interact with bacterial membranes and increase membrane permeability, thereby causing changes in membrane structure and inducing DNA damage and enzyme inactivation, which eventually leads to cell death [18,87,88,89]. The efficiency of AgNP-mediated cytotoxicity depends on size, stability, and AgNP concentration in the growth medium. Effectiveness also depends on the bacterial species and is particularly influenced by bacterial cell wall structure, membrane composition, and membrane-associated properties [90].

## 3. Materials and Methods

### 3.1. Materials

Luria–Bertani (LB) agar and nutrient agar was purchased from USB Corporation (Santa Clara, CA, USA). Brain Heart Infusion agar, Mueller Hinton Broth (MHB) or Mueller Hinton Agar, silver nitrate, quercetin dihydrate, and crystal violet were purchased from Sigma-Aldrich (St. Louis, MO, USA). DTNB (5,5′-dithio-bis-(-2-nitrobenzoic acid)), and glutathione (GSH) were purchased from Sigma-Aldrich (St. Louis, MO, USA). LB agar was purchased from USB Corporation (Santa Clara, CA, USA). The in vitro toxicology assay kit was purchased from Sigma-Aldrich (St. Louis, MO, USA). BacTiter-Glo™ Microbial Cell Viability Assay Reagent was purchased from Promega (Madison, WI, USA). All other chemicals were purchased from Sigma-Aldrich (St. Louis, MO, USA) unless otherwise stated.

### 3.2. Synthesis and Characterization of AgNPs

AgNPs synthesis was performed using quercetin according to a previously described method [24]. First, 0.5 mg/mL quercetin was dissolved in DMSO (dimethylsulfoxide) mixed with 1 mM AgNO_3_, and the resulting mixture was incubated at 40 °C for 1 h. Bio-reduction of the silver ions was monitored spectrophotometrically at 420 nm. Further characterization of the synthesized AgNPs was performed as previously described [24].

### 3.3. Milk Sampling

Milk samples (*n* = 100) from 100 lactating dairy goats from the southern region of China were collected in sterile containers. Milk samples testing positive based on WST were transported to the lab at 4 °C. Udders of lactating goats were observed visually and through palpation for presence of lesions and inflammatory signs (i.e., heat, pain, redness, and swelling) and loss of function. The udder was washed with warm tap water and soap and then dried completely. Teats were disinfected with a piece of cotton wool soaked in 70% ethyl alcohol. Goats were not treated with systemic or intra-mammary antimicrobial agents during the lactation period before enrollment in this study. All collected samples were tested using WST.

### 3.4. Bacterial Characterization

Sampling and characterization of bacteria was performed as described earlier [91]. Each milk sample (0.5 mL) was cultured on brain heart infusion agar using spreading technique and plates incubated at 37 °C for 24 h. Bacterial colonies with different colony characteristics were isolated and purified via multiple streaking. Purified bacterial isolates were biochemically characterized using appropriate selective/differential media and fermentation of different sugars following the identification flow charts provided in Bergey’s Manual for Determinative Bacteriology [91]. Bacterial identification was performed based on pooled observations of colony characters, microscopic morphology, and biochemical profiles.

### 3.5. Isolation of MDR Bacteria

Antimicrobial resistance patterns were determined by the Kirby–Bauer disk diffusion test performed according to recommendations (CLSI 2013) and also previously described by Franca et al. [92]. We used the following antibiotics: streptomycin (10 μg), enrofloxacin (5 μg), norfloxacin (10 μg), ciprofloxacin (5 μg), amoxicillin (10 μg), oxacillin (1 μg), doxycycline (30 μg), tetracycline (30 μg), erythromycin (15 μg), lincomycin (2 μg), rifampicin (05 μg), amikacin (30 μg), gentamicin (10 μg), tobramycin (10 μg), ciprofloxacin (5 μg), cefazolin (30 μg), cephalothin (30 μg); ceftazidime (30 μg), erythromycin (15 μg), ampicillin (10 μg), tetracycline (30 μg), lincomycin (2 μg), clindamycin (2 μg), imipinem (10 μg), meropenem (10 μg), bacitracin (10 μg), trimethoprim-sulfamethoxazole (SXT 1.25/23.75 μg), chloramphenicol (C30 μg), methicillin (5 μg), and vancomycin, (30 μg). The multidrug resistance rate (MDR rate) was calculated by dividing by the total number of antimicrobial resistant groups [93]. The inhibition zone diameter around each disk was measured and was interpreted as described in the NCCLS (2013). Bacteria exhibiting resistance to more than two classes of antibiotics were checked for sensitivity against different combinations of antibiotics. *Pseudomonas* and *Staphylococcus* species were observed to be the most abundant microorganisms present in the milk samples and also showed resistance to several antibiotics. Therefore, *Pseudomonas* and *Staphylococcus* were selected for further analysis. To identify dominant bacterial species like *Pseudomonas species* and *Staphylococcus* species, 16s rRNA gene sequence analysis was performed according to a previously described method [94].

### 3.6. Bacterial Strains and Growth Conditions

Bacterial growth and media preparation were performed according to a previously described method described [16]. Briefly, all the cultures were first grown aerobically at 37 °C in MHB media. Cultures were maintained by streaking a bacterial colony in MHB agar plates and sub-culturing every fortnight. Pure colonies were isolated and stored at −80 °C. All test strains were grown and maintained in MHB medium. Cells were grown and harvested by centrifugation at 6000 rpm for 10 min and then resuspended in sterile MHB medium until an optical density at 600 nm (OD_600_) of 1.0 was reached.

### 3.7. MIC and MBC Determination

AgNPs susceptibility tests were carried out in 96-well microtiter plates using a standard two-fold broth microdilution of the antibacterial agents in MHB following the Clinical and Laboratory Standards Institute (CLSI) guidelines (CLSI, 2003). To determine the MICs of AgNPs, all the test strains were exposed to 0–100 µg/mL AgNPs. AgNPs solutions were prepared using phosphate-buffered saline (PBS) and tested for antibacterial efficacy. The appropriate AgNPs concentration and 1 mL of the bacterial suspension were mixed in MHB media to obtain a final bacterial concentration of 10^5^–10^6^ colony forming units (CFUs)/mL and incubated for 24 h. After treatment, 100 μL of the reaction mixture was diluted to 1 mL, and 100 μL of the total mixture was used for plating. Loss of cell viability was determined using the colony counting method. 

### 3.8. Antimicrobial Activity of AgNPs

Assessment of AgNPs microbial toxicity was performed as previously described [16]. To examine the effects of AgNPs on growth of the isolates, overnight cultures were centrifuged at 6000 rpm for 5 min, washed with 1× PBS, and the pellet was resuspended in saline buffer. Finally, the OD_600_ of the sample was adjusted to 0.1. Cells (5 × 10^5^ cells in 96-well round-bottom plates in triplicate) were exposed to different concentrations of AgNPs. Bacteria were harvested at the indicated time points or dose responses, and the number of CFUs was counted. Media only and media containing AgNPs were used as controls. All samples were plated in triplicate, and values were expressed as the average of three independent experiments.

### 3.9. In Vitro Cytotoxicity Assays

In vitro cytotoxicity assays were performed as described previously [16] with suitable modifications. Cells were grown overnight in MHB broth at 37 °C and regrown in fresh medium for 24 h before centrifugation and suspension in deionized water. A cell suspension consisting of 10^6^ cells/mL was incubated with various concentrations of AgNPs for 24 h at 37 °C. After incubation, bacteria were harvested at the indicated time points, and 100-μL aliquots were taken from each sample to determine the number of CFUs. The experiment was performed with various controls, including a positive control (AgNPs and MHB media without inoculum) and a negative control (MHB and inoculum without AgNPs). All samples were plated in triplicate, and values were averaged from three independent experiments.

### 3.10. Bacterial Cell Lysate Preparation

To prepare bacterial cell lysates, cells were grown and centrifuged at 4 °C for 10 min at 5000 rpm. The resulting pellet was washed with PBS and resuspended in bacterial lysis buffer, followed by the addition of lysozyme and incubation at 4 °C for 4 h before probe sonication for 5 min. Cell debris were removed by centrifugation at 10,000 rpm, and the supernatant was collected and used for enzyme assays [16].

### 3.11. Assay for the Leakage of Proteins and Reducing Sugars

Protein leakage from bacterial cells was determined as described previously, and the amount of sugar leakage was determined according to a previously described method [16,21]. The AgNPs concentration was adjusted to the desired level for each isolate, and the concentration of bacterial cells was 10^6^ CFUs/mL. Each culture was incubated in a shaking incubator at 37 °C for 4 h. Culture samples (1 mL) were centrifuged at 4 °C for 30 min at 10,000 rpm, and the supernatant was frozen at −20 °C before estimation of protein and sugar levels.

### 3.12. Measurement of LDH Activity

LDH activity was determined by measuring the reduction of NAD^+^ to NADH and H^+^ during the oxidation of lactate to pyruvate according to a previously described method [45,52]. Bacterial cells were adjusted to 10^6^ CFUs/mL. Each culture was incubated in a shaking incubator at 37 °C for 12 h. AgNPs concentrations were adjusted to the respective MIC concentration for each bacterium. After incubation with AgNPs, the culture was centrifuged at 4 °C for 30 min at 300× *g*, and the supernatant was discarded. The pellet was washed twice and then treated with LDH reaction solution in a microplate [52]. The plate was then incubated with gentle shaking on an orbital shaker for 30 min at room temperature. After incubation, the O.D. of the plate was measured at 490 nm.

### 3.13. Measurement of ATP Levels

Measurement of ATP levels in the bacterial culture supernatant was conducted according to the methods of Mempin et al. [55], Zhang et al. [95], and the manufacturer’s instructions for BacTiter-Glo™ Microbial Cell Viability Assay Reagent (Promega, Madison, WI, USA). In the luciferase-based assay, ATP levels were determined by measuring luminescence levels and comparing against an ATP standard curve. Briefly, 100 µL of culture supernatant from the control or treated cells was mixed with an equal volume of BacTiter-Glo™ Microbial Cell Viability Assay Reagent in a 96-well opaque plate and incubated at room temperature for 5 min. After incubation, luminescence was read using a SpectraMax M2 plate reader (Molecular Devices, Sunnyvale, CA, USA).

### 3.14. Measurement of Reactive Oxygen Species (ROS) Levels

ROS generation was measured according to the previously described method using 2′,7′-dichlorofluorescein diacetate (DCFDA) [52,95]. Bacterial cells (10^6^ CFU/mL) were treated with or without AgNPs at the required temperature for 12 h. After incubation, cells were centrifuged at 4 °C for 30 min at 300× *g*, after which the supernatant was treated with 100 μM DCFDA for 1 h. The amount of ROS produced in the sample was detected at the excitation wavelength of 485/20 nm of fluorescence excitation and emission wavelength of 528/20 nm using a fluorescence multi-detection reader (BIOTEK, Winooski, VT, USA).

### 3.15. MDA Measurements

Cells grown in culture media were incubated at the required temperature, and MDA levels were determined using thiobarbituric acid-reactive substances assay as previously described previously suitable modifications [23,95,96]. Briefly, 1 mL of culture medium of AgNPs-treated cells were added with 10% SDS and swirled vigorously. Next, the resulting mixture was added with 2 mL of freshly prepared thiobarbituric acid (TBA) and incubated at 95 °C for 60 min. The reaction was allowed to cool at room temperature and centrifuged at 5000 rpm for 10 min. The OD of the supernatant was measured at 530 nm.

### 3.16. Estimation of GSH Levels

For enzymatic determination of GSH levels, cells were incubated at required temperature with or without AgNPs for 12 h. Cells were pelleted centrifugation at 10,000 rpm for 5 min, washed with PBS, and lysed. The lysate was prepared as described above. GSH levels in the clear supernatant were determined based on the reduction of 5,5′-dithiobis-(2-nitrobenzoic acid) by the GSH reductase system as previously described [95]. All samples were prepared in triplicate. DTNB (Ellman’s reagent, 5,50-dithio-bis-(2-nitrobenzoic acid) Sigma-Aldrich) was added to the mixtures to yield a yellow product. The absorbance at 412 nm was measured using a spectrophotometer.

### 3.17. Determination of GST Total Activity

GST activity was determined as previously described [95] with suitable modifications. GST was assayed spectrophotometrically at 37 °C in a mixture containing 900 mL of 100 mM potassium phosphate buffer (pH 6.5), 25 mL of 40 mM 1-chloro-2,4-dinitrobenzene, 50 mL of 1 mM GSH, and 25 μL of enzyme extract. The reaction mixture was monitored by measuring the increase in absorbance at 340 nm for 5 min. GST activity was expressed as μmol/min/mg protein.

### 3.18. Determination of Superoxide Dismutase and Catalase Activity

Catalase activity was measured using a previously described protocol [95] with appropriate modifications. A typical reaction contains a mixture of 500 μL 0.1 M phosphate buffer (pH 7.5) and 500 μL of freshly prepared 0.9% H_2_O_2_ solution. The resulting mixture was added with 100 µL of bacterial cell lysate and incubated for 3 min. The reaction was terminated by drop wise addition of 2 N H_2_SO_4_. The unreacted H_2_O_2_ was titrated with 0.1 N KMnO_4_. Boiled bacterial cell lysate with or without AgNPs was used as a blank. Catalase activity was calculated as previously described. SOD activity was measured as previously described [95] using an SOD Assay Kit (Sigma-Alrich, 19160). Specific enzyme activity was calculated from the enzyme activity and total protein content in the bacterial cell lysate as determined using the Bradford method [27].

### 3.19. Statistical Analysis

All experiments were carried out in triplicate and repeated at least three times. The results are presented as means ± SD. All experimental data were compared using the student’s *t*-test. *p* < 0.05 was considered statistically significant.

## 4. Conclusions

Bacterial infections cause increased morbidity and mortality both in humans and animals. Widespread and frequent use of antibiotics has led to the emergence of various multidrug-resistant bacterial pathogens. The growing concern regarding multidrug-resistant bacterial strains has resulted in an urgent need for the development of alternative, cost effective, simple, and environmentally friendly approach. AgNPs have been recognized as more effective antibacterial agents compared to other metal nanoparticles. Therefore, we designed a nanomaterial-based antimicrobial therapy against *P. aeruginosa* and *S. aureus* strains isolated from milk samples from mastitis-infected goats. Herein, we demonstrate successful biomolecule-assisted synthesis of AgNPs using quercetin. The synthesized AgNPs exhibit distinct spherical shapes with an average size of 11 nm. Results from the present study suggest that the biologically synthesized AgNPs were effective against the tested bacteria. AgNP-induced loss of cell viability was mediated via mechanisms, such as the impairment of metabolic activity, leakage of intracellular proteins and sugars, and increased oxidative stress, which consequently result in imbalance in the antioxidant proteins. AgNPs exert antibacterial effects through loss of membrane stability and inactivation of respiratory chain dehydrogenases, and eventual ROS generation, which inhibit respiration and growth of cells. The use of biologically synthesized AgNPs represents a suitable and alternative strategy for treatment of infections caused by MDR bacteria in dairy animals. In addition, AgNP-based antimicrobial agents can overcome the limitations of conventional antimicrobial agents. Finally, this study is the first to provide evidence for the mode of action of AgNPs against MDR pathogens isolated from subclinical mastitis-infected goats.

## Figures and Tables

**Table 1:** Size and ζ potential of AgNPs in water and MHB media.

Measurement	Water	MHB Media
Size (nm)	11	20
ζ potential (mV)	25.5	37.7

**Table 2:** MIC value of AgNPs.

Strain	Organism	MIC (µg/mL)	MBC (µg/mL)
MDR12	*P. aeruginosa*	1	2
MDR13	*S. aureus*	2	4
